# Encoding a Melody Using Only Temporal Information for Cochlear-Implant and Normal-Hearing Listeners

**DOI:** 10.1177/2331216517739745

**Published:** 2017-11-22

**Authors:** Ann E. Todd, Griet Mertens, Paul Van de Heyning, David M. Landsberger

**Affiliations:** 1 Department of Otolaryngology, New York University School of Medicine, NY, USA; 2 Department of Otorhinolaryngology, Head and Neck Surgery, Antwerp University Hospital, University of Antwerp, Belgium

**Keywords:** temporal, pitch, cochlear implant, music, apex

## Abstract

One way to provide pitch information to cochlear implant users is through amplitude-modulation rate. It is currently unknown whether amplitude-modulation rate can provide cochlear implant users with pitch information adequate for perceiving melodic information. In the present study, the notes of a song were encoded via amplitude-modulation rate of pulse trains on single electrodes at the apex or middle of long electrode arrays. The melody of the song was either physically correct or modified by compression or expansion. Nine cochlear implant users rated the extent to which the song was out of tune in the different conditions. Cochlear implant users on average did not show sensitivity to melody compression or expansion regardless of place of stimulation. These results were found despite the fact that three of the cochlear implant users showed the expected sensitivity to melody compression and expansion with the same task using acoustic pure tones in a contralateral acoustic ear. Normal-hearing listeners showed an inconsistent and weak effect of melody compression and expansion when the notes of the song were encoded with acoustic pulse rate. The results suggest that amplitude-modulation rate provides insufficient access to melodic information for cochlear-implant and normal-hearing listeners.

## Introduction

Although cochlear implants (CIs) allow many users to understand speech in quiet, performance on difficult tasks such as speech perception in noise (e.g., Oxenham & Kreft, 2014; Stickney, Zeng, Litovsky, & Assmann, 2004) and music perception suffers (e.g., Gfeller et al., 2005; Kong, Cruz, Jones, & Zeng, 2004). Presumably one of the reasons these tasks are difficult is that pitch information is poorly perceived by CI users (Crew, Galvin, Landsberger, & Fu, 2015; Gfeller et al., 2007; Green, Faulkner, & Rosen, 2004; Sucher & McDermott, 2007). Normal-hearing (NH) listeners use the fundamental frequency of a speaker to segregate that speaker from other interfering sounds (Darwin, Brungart, & Simpson, 2003; Drullman & Bronkhorst, 2004). In addition, shifts in the fundamental frequency indicate emotion (Jiam, Caldwell, Deroche, Chatterjee, & Limb, 2017; Luo, Fu, & Galvin, 2007; Williams & Stevens, 1972) as well as whether a sentence is a statement or a question (O’Shaughnessy & Allen, 1983). In tonal languages, shifts in the fundamental frequency indicate the meaning of words (e.g., Luo & Fu, 2004; Xu, 1994). Therefore, improving pitch perception through CIs is important for improving speech understanding in complex situations. Similarly, improved pitch perception should greatly improve music perception for CI users.

For CI stimulation, the place of stimulation (i.e., electrode) and rate of stimulation are independent parameters. While keeping the temporal information fixed, changing the electrode that is stimulated is perceived as change in pitch or brightness (an aspect of timbre; Eddington, Dobelle, Brackmann, Mladejovsky, & Parkin, 1978; H. J. McDermott, 2004; Shannon, 1983; Townshend, Cotter, Van Compernolle, & White, 1987). Similarly, changing the pulse rate or amplitude-modulation rate at a fixed electrode also provides a change in pitch (Eddington et al., 1978; Fearn & Wolfe, 2000; Zeng, 2002). When combined, changes in rate and place both affect pitch (e.g., Landsberger, Vermeire, Claes, Van Rompaey, & Van de Heyning, 2016; Luo, Padilla, & Landsberger, 2012; Stohl, Throckmorton, & Collins, 2008). However, the perceptual quality of a change in rate and a change in place are different and independent (Landsberger et al., 2016; McKay, McDermott, & Carlyon, 2000; Tong, Blamey, Dowell, & Clark, 1983). For NH listeners, the temporal aspects of a stimulus can be studied independently of the place of stimulation by using amplitude-modulated stimuli such as complex tones with only unresolved components (Bernstein & Oxenham, 2003; Carlyon & Deeks, 2002; Carlyon & Shackleton, 1994; Houtsma & Smurzynski, 1990). The presentation of complex tones filtered to have only unresolved components around a spectral peak to NH listeners is analogous to the presentation of low-rate electrical pulse trains (or high-rate amplitude-modulated pulse trains) with single electrodes to listeners with CIs (Carlyon & Deeks, 2002; Majdak, Laback, & Baumgartner, 2006).

Clinical CI sound-processing strategies allow CI users to hear higher frequency sounds as being higher in pitch than lower frequency sounds, provided the frequency differences are large enough (e.g., Crew et al., 2015; Galvin, Fu, & Nogaki, 2007; Gfeller et al., 2007). We refer to the ability to rank pitches in the correct order as *ordinal* pitch. This has also been referred to as contour perception (Dowling & Fujitani, 1971). Ordinal pitch may be adequate for perception of speech intonation and melodic contour. However, ordinal pitch by itself does not indicate that the listener perceives the ratio between frequencies, which is important for melody perception. We refer to the ability to perceive the ratio between frequencies as *rational* pitch, which has also been referred to as interval perception (Luo, Masterson, & Wu, 2014; Pijl & Schwarz, 1995b). For example, when a frequency is doubled, correct rational pitch would be equivalent to the perception of an octave change in pitch. If a listener does not have rational pitch, they will not perceive melodic information. If a listener has rational pitch but does not have correct rational pitch, melodies will sound out of tune. Findings suggest that with clinical sound processing strategies, correct rational pitch is not maintained, as CI users perceive melodies as sounding more out of tune than NH listeners (Luo et al., 2014). The present study further examines melody perception of listeners with CIs; however, without the clinical processor such that the place and temporal aspects of the stimulation are highly controlled.

Place pitch with CIs is coded by stimulation of different electrodes in the cochlea, each of which is typically programed to represent a different band of frequencies. Modern CI sound-processing strategies are unlikely to preserve correct rational pitch through place coding, since the relationship between frequencies presented to a CI processor and where they are represented on the cochlea is distorted and varied across listeners depending on the electrode array placement and frequency allocation (Landsberger, Svrakic, Roland, & Svirsky, 2015). However, even if the appropriate relationships between frequency and place were maintained, the spread of excitation produced by each electrode is broad which likely limits rational place pitch (Abbas, Hughes, Brown, Miller, & South, 2004; Oxenham, 2008). Broad spread of excitation may make it such that stimulation of a single electrode is unlikely to sound like a pure tone. Furthermore, broad spread of excitation with multielectrode stimulation would result in unresolved harmonics limiting rational place pitch for complex tones.

Temporal coding of pitch is also provided by modern CI strategies. In envelope-based encoding strategies such as Continuous Interleaved Sampling (CIS), Advanced Combination Encoder (ACE), and HiResolution (HiRes) (Firszt, Holden, Reeder, & Skinner, 2009; Loizou, 2006; Wilson et al., 1991), the temporal envelope of electrical pulse trains is often modulated at the fundamental frequency of the signal. Temporal fine-structure strategies (e.g., FSP or FS4 or FS4p) specifically encode temporal information by providing packets of pulses at waveform zero-crossings on apical electrodes while providing temporal coding via envelope modulations on the remaining electrodes (Riss et al., 2014). The upper limit for temporal pitch discrimination is approximately 300 Hz depending on the listener and site of stimulation (Eddington et al., 1978; Kreft, Oxenham, & Nelson, 2010; Landsberger & McKay, 2005; Shannon, 1983; Tong et al., 1983), suggesting that at least for low rates, there is the possibility that temporal coding may provide CI users with rational pitch. Rate discrimination of CI users is poorer than complex-tone discrimination of NH listeners (Moore & Peters, 1992; Zeng, 2002), which may limit the usefulness of temporal coding for rational pitch. This is the case because the ability to discriminate two different rates is necessary for perceiving the ratio between the rates. Despite limitations in rate discrimination, it has been found that in some cases, CI users show fairly accurate musical interval perception. Pijl and Schwarz (1995b) found that three CI users could identify musical intervals encoded by pulse rates on single electrodes at base rates from 127 to 163 pulses per second (pps) with average performance typically within one semitone of the expected interval. These same three listeners could also adjust pulse rates to specific musical intervals at a base rate of 93 pps with average performance typically within one semitone of the expected interval (Pijl & Schwarz, 1995a). These findings suggest that some CI users may be able to use temporal coding for perception of melodies. Marimuthu, Swanson, and Mannell (2016) found that CI users were able to use temporal coding (pulse rate) to identify backward melodies and to identify heavily warped melodies compared with physically correct melodies. However, the extent to which the CI users in that study relied on rational pitch is unclear. To identify the backwards melodies, listeners could rely on the ordinal relationships between the notes of the melodies. This was not the case for the warped melodies; however, the listeners may have been relying on the absence of intermediate-sized intervals (i.e., the presence of only relatively small and large intervals) with the more heavily warped melodies. Awareness of the absence of intermediate-sized intervals might not require rational pitch if listeners have an ability to perceive the general size of intervals that does not depend on rational pitch. Thus, it remains unclear the extent to which CI users can use temporal coding for melody perception. The present study investigated whether listeners with CIs show rational pitch with temporal coding.

A number of studies have investigated the effect of place of stimulation in the cochlea on the use of temporal information. It has been suggested that auditory nerve fibers with low characteristic frequencies may be better able to encode temporal information than fibers with high characteristic frequencies. If this is the case, there may be an advantage to stimulation of nerve fibers with low characteristic frequencies for melody perception with temporal coding. Middlebrooks and Snyder (2010) found evidence to support this idea in an animal model, which showed higher rates of phase locking of neurons in the inferior colliculus when auditory nerve fibers with characteristic frequencies below 1.5 kHz were stimulated electrically (Middlebrooks & Snyder, 2010). For humans, auditory nerve fibers with low characteristic frequencies are largely located deeper than the first cochlear turn. However, most electrode arrays, including those of the participants examined by Marimuthu et al. (2016), are not designed to be inserted past the first cochlear turn (Landsberger et al., 2015). Marimuthu et al. (2016) found no advantage for melody perception from stimulation at the apical end of the electrode array and found a detriment in performance for identifying backwards and warped melodies with apical stimulation for rates above 263 pps. However, it is possible that no advantage was found from stimulation at the apical end of the electrode array in that study because the most apical electrodes were not positioned apically enough (i.e., into the second cochlear turn) to stimulate fibers with low characteristic frequencies.

A few studies have investigated listeners’ use of temporal coding using electrode arrays designed to be inserted 1.5 to 1.75 turns into the cochlea (Baumann & Nobbe, 2004b; Kong, Deeks, Axon, & Carlyon, 2009; Landsberger et al., 2016; Stahl, Macherey, Meunier, & Roman, 2016). Neither Baumann and Nobbe (2004b) nor Kong et al. (2009) found an advantage of apical stimulation over basal stimulation for accuracy in rate discrimination; however, neither study examined performance with the most apical electrode of the electrode array. In contrast, Stahl et al. (2016) found better pulse rate difference limens when stimulating with the most apical electrode. Notably though, only very low rates (≤104 pps) were examined. Nevertheless, this finding suggests that there may be an advantage to apical stimulation with temporal coding, and this advantage may extend to melody perception. In addition, Landsberger et al. (2016) found evidence of better sound quality with apical stimulation in that listeners scaled low-rate pulse trains as sounding cleaner with stimulation of the most apical electrodes. Potentially, cleaner sound quality would lend itself to better melody perception.

In the present study, we investigated whether temporal coding with CIs can provide rational pitch for melody perception by examining listeners’ perception of a familiar melody (Happy Birthday) encoded with amplitude-modulation rate. This was done by examining listeners’ ability to identify when the melody was out of tune to various extents. In addition, we investigated whether the use of temporal coding for melody perception depends on cochlear location. This was done to further investigate whether there is an advantage of apical stimulation for melody perception with temporal coding. We hypothesized that the use of temporal coding for rational pitch may be better with apical stimulation, as this is the region where low frequency information is encoded in the normal auditory system.

## Methods

### Participants

**Figure 1.**
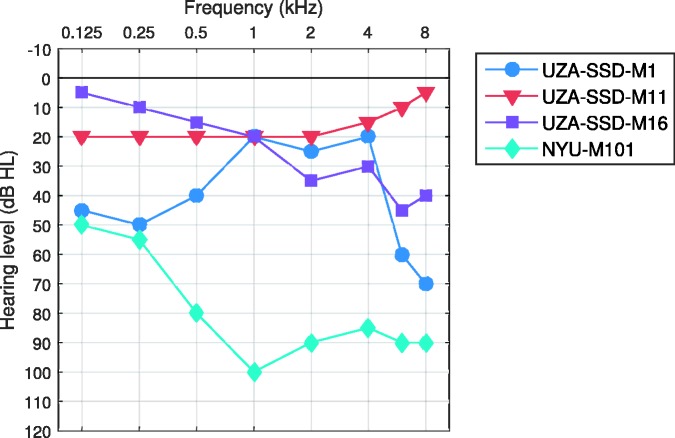
Pure-tone audiograms for the four participants with contralateral CIs.

Nine CI users participated in this study. Table 1 shows the characteristics of the listeners with CIs. Table 2 shows the CI participants’ responses to questions about their music experience. The music experience questions were obtained from D. R. Friedmann (personal communication, June 1) and were a modification of the questionnaire used by Gfeller et al. (2000). All CI participants indicated that they were familiar with the melody of the song Happy Birthday prior to the experiment. Seven of the CI users were tested at the Antwerp University Hospital (UZA) and two were tested at New York University (NYU) as indicated in the participant codes. All were users of CIs manufactured by MED-EL and had electrode arrays which were 31 mm in length except participant NYU-M101 who had a 28-mm electrode array. Information on electrode insertion depths was not available for the participants. However, the 20% trimmed mean insertion depth was 624° for CI users with 31-mm electrode arrays implanted at the same hospital and by the same surgeon as the seven CI users tested at UZA in the present study. It is therefore likely that the insertion depths for our participants were similar. It is worth noting that this estimate is typical of what has been found in other studies. Landsberger et al. (2015) found that the 20% trimmed mean insertion depth was 627° across eight additional studies with 31-mm electrode arrays (Baumann & Nobbe, 2004a; Gani, Valentini, Sigrist, Kos, & Boex, 2007; Hamzavi & Arnoldner, 2006; Kos, Boex, Sigrist, Guyot, & Pelizzone, 2005; Landsberger et al., 2015; Radeloff, Mack, Baghi, Gstoettner, & Adunka, 2008; Trieger, Schulze, Schneider, Zahnert, & Murbe, 2011; Vermeire et al., 2008). The mean insertion depth across three studies for the 28-mm electrode array was 571° (Boyer et al., 2015; Franke-Trieger, Jolly, Darbinjan, Zahnert, & Murbe, 2014; O’Connell et al., 2016). Four of the CI users had usable acoustic hearing in their contralateral ears, the pure-tone audiograms of which are shown in Figure 1. Participants UZA-SSD-M11 and UZA-SSD-M16 had NH in the relevant range (i.e., 90–340 Hz) in their contralateral ears. Participants UZA-SSD-M1 and NYU-M101 had hearing loss in their contralateral ears. Participant NYU-M101 typically wore a hearing aid in his acoustic ear but was tested without his hearing aid in the present study.

**Table 1.** Characteristics of the Participants With CIs.

Participant	Age (years)	CI Ear	Age at onset of hearing loss (years)	Duration of CI use (years)	Etiology	Implant	Electrode array	Clinical strategy
UZA-SSD-M1	64	Left	56	5	Meniere’s	SonataTI	FLEXsoft	FS4
UZA-SSD-M11	33	Right	21	10	Viral	Pulsar	FLEXsoft	FS4
UZA-SSD-M16	68	Left	55	9	Sudden deafness	Pulsar	FLEXsoft	FSP
NYU-M101	24	Right	0.5	0.22	Unknown	Synchrony	Flex28	FS4
NYU-M102	68	Right	45	0.5	Unknown	Synchrony	Standard	FSP
UZA-M13	32	Right	15	6	Unknown	SonataTI	FLEXsoft	FS4
UZA-M10	53	Right	10	9	Unknown	Pulsar	FLEXsoft	FS4
UZA-M16	53	Right	<6	5	Meningitis	SonataTI	FLEXsoft	FSP
UZA-M17	54	Left	42	3	Meniere’s	Concerto	FLEXsoft	FS4

CI = cochlear implant.

**Table 2.** Music Experience of the Participants With CIs.

Participant	Q1 (hours/week)	Q2 (hours/week)	Q3 (hours/week)	Q4 (years)	Q5 (years)
UZA-SSD-M1	>10	>10	>10	never	never
UZA-SSD-M11	2–5	never	2–5	never	never
UZA-SSD-M16	>10	0–2	>10	never	2–5
NYU-M101	NA	0–2	0–2	5–10	5–10
NYU-M102	8	never	5–10	5–10	never
UZA-M13	5	2–5	5–10	>10	2–5
UZA-M10	never	never	never	never	never
UZA-M16	5–10	5–10	5–10	never	never
UZA-M17	>10	2–5	never	2–5	0–2

Q1: Did you listen to music BEFORE hearing loss?

Q2: Did you listen to music AFTER hearing loss (before implantation)?

Q3: Do you listen to music with your cochlear implant?

Q4: Have you ever taken music lessons on instrument or voice?

Q5: Did you ever participate in music ensembles (band, orchestra, choir)?

In addition to the listeners with CIs, there were 10 NH listeners. All NH listeners had pure-tone detection thresholds ≤25 dB HL from 125 Hz to 8 kHz in octave steps in the ear used for testing. NH listeners ranged in age from 21 to 47 years of age (Mean = 30.9, *SD* = 8.6). All NH listeners were tested at NYU. This research was approved by the New York University School of Medicine institutional review board (Assurance Number 00004952) and the ethics committee (12/10/94) at the UZA. Informed consent was obtained from participants prior to the study, and a modest financial reimbursement was provided for participation.

### Equipment

Electric stimuli were presented using the MAX interface developed by MED-EL and were generated using custom-written software in MATLAB and the RIB2 DLL provided by the University of Innsbruck. Acoustic stimuli were presented using an Edirol UA-25 sound card and Sony MDR-7506 headphones.

### Electric Stimuli

The electric stimuli consisted of sinusoidally amplitude-modulated trains of biphasic pulses presented in monopolar mode with each phase having opposite polarity. Pulses were presented at 2000 pps with a 50-µs phase duration and a 2.1-µs interphase gap. Stimuli were presented to individual electrodes numbered 1, 2, 3, 7, and 9, where lower numbered electrodes have more apical locations. Pulse trains had an amplitude-modulation depth of 75% of the maximum level (i.e., 12 dB from the maximum). Seventy-five percent was chosen as it would likely extend through a large portion of the listeners’ dynamic ranges, which typically range from 6 to 17 dB (Kreft, Donaldson, & Nelson, 2004). Maximum levels corresponded to levels that produced most comfortable loudness with the stimuli used for loudness mapping and balancing. Stimuli used for loudness mapping and balancing were 500-ms pulse trains with an amplitude-modulation rate of 200 Hz. Stimuli for the experimental task consisted of sequences of 25 pulse trains with 50-ms interpulse-train intervals, except in the case of participant UZA-SSD-M16, who was the first participant tested and listened with 10-ms interpulse-train intervals. Interpulse-train intervals of 10 ms were originally included to approximate the perceptual gap produced by the 10-ms onset and offset ramps used with the pure-tone stimuli (described later). However, the interpulse-train intervals were changed to 50 ms because the second participant perceived the song as one continuous stream of sound with the 10-ms intervals. The 50-ms interpulse-train intervals slowed the melody down and likely facilitated the listeners hearing the rhythm of the song. No ramping was used for the electric stimuli. Each sequence consisted of pulse trains with durations equal to 500 ms × relative durations, where *relative durations* = (.75, .25, 1, 1, 1, 2, .75, .25, 1, 1, 1, 2, .75, .25, 1, 1, 1, 1, 1, .75, .25, 1, 1, 1, 2) and corresponded to the rhythm of the song Happy Birthday. The modulation rates (f_m_) of the pulse trains within each sequence were equal to f_m_ = root note × (2^(1/12)^) ^(semitone exponent×steps)^. *Steps* corresponded to the number of semitones between the root (lowest) note and the other notes of the song Happy Birthday and was equal to (0, 0, 2, 0, 5, 4, 0, 0, 2, 0, 7, 5, 0, 0, 12, 9, 5, 4, 2, 10, 10, 9, 5, 7, 5). *Root note* was the frequency of the lowest note of the sequence and was equal to 90 or 110 Hz, except with participant UZA-SSD-M16 who was tested at 70- and 90-Hz root notes. *Semitone exponent* determined the expansion (semitone exponent > 1) or compression (semitone exponent < 1) of the melody and was equal to .43, .63, .83, 1, 1.23, 1.43, or 1.63. The modulation rates were never greater than 340 Hz. The song Happy Birthday was chosen as it is well known in both the United States and Belgium. However, as it is only one song, the results do not necessarily generalize to other songs.

### Pure-Tone Stimuli

Acoustic pure-tone stimuli were created using a 44.1 kHz sampling frequency and a 16-bit depth resolution. Similar to the electrical stimuli, pure tones were concatenated to form 25-note sequences which formed the song Happy Birthday. The same semitone exponents and root notes (90, 110 Hz) that were used with the electric stimuli were used with the pure-tone stimuli. The durations of the pure tones matched those of the electric pulse trains. Ten-ms Hann on- and off-ramps were used for each pure tone. The interpure-tone interval was 0 ms. Pure tones were normalized using the root-mean-square amplitude. The pure-tone stimuli were tested at a comfortable listening level in the acoustic ears of the four CI users with usable acoustic hearing. Amplitudes were not adjusted in a frequency-dependent manner to make up for acoustic hearing losses. There was no attempt to loudness balance the electric and the acoustic stimuli for these listeners. Instead, both sets of stimuli were at a level the participant determined to be most comfortable. The pure tones were presented monaurally at 78 dB SPL to the NH listeners.

### Acoustic Pulse-Train Stimuli

**Figure 2.**
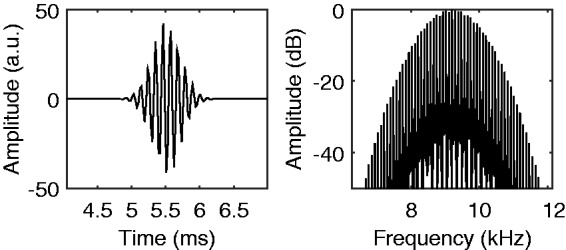
Acoustic pulsatile stimuli used with the NH participants. A single acoustic pulse (left panel) and spectrum of an acoustic pulse train (right panel). The stimuli had a 9200-Hz center frequency and a rate of 90 Hz.

In addition to the pure-tone stimuli, NH listeners were tested with acoustic pulsatile stimuli. The pulse rates and durations of the acoustic pulse trains matched the amplitude-modulation rates and durations of the electric pulse trains. The same semitone exponents and root notes (90, 110 Hz) that were used with the electric stimuli were used with the acoustic pulse-train stimuli. Gaussian-shaped pulses were created in the time domain using the formula specified in Goupell, Majdak, and Laback (2010). Each pulse train was created by summing together a series of equal-amplitude pulses with peaks at time points spaced out by the period of the pulse rate. An integer number of pulses was always used for each pulse train. Pulse trains were concatenated with 50-ms interpulse-train intervals. No ramping was used for the acoustic pulsatile stimuli. Subsequently, the sequence of pulse trains was multiplied by either a 6500-Hz or 9200-Hz sine wave such that the pulses were an amplitude modulation of those frequencies, and the pulse trains were spectrally centered at those frequencies. These high center frequencies were chosen so that the stimuli would be unresolved by the NH auditory system (Carlyon & Deeks, 2002). Pulses to be presented at 6500 Hz and 9200 Hz had equivalent rectangular bandwidths of 1380 Hz and 1940 Hz, respectively. These values were used because they produced bandwidths with equal amounts of spreads of excitation (1.5 mm) according to the Greenwood function (Greenwood, 1990). Figure 2 shows the waveform of a pulse and the spectrum of the first note at 90 Hz. The sequence of pulse trains was root-mean-square amplitude normalized and was presented monaurally at 65 dB SPL. Pink noise was presented from 0 Hz to 22.05 kHz along with the acoustic pulsatile stimuli to mask combination tones. The pink noise started and finished 150 ms before and after the pulsatile stimuli, respectively, and was ramped on and off using 50-ms Hann windows. The pink noise was presented monaurally in the same ear as the pulse trains at 60 dB SPL.

## Procedure

### Loudness Mapping and Balancing

To find most comfortable loudness levels for the electric stimuli, maximum current amplitudes of 500-ms pulse trains with an amplitude-modulation rate of 200 Hz were raised in 5-µA steps. The levels that the listener indicated were most comfortable were recorded. To loudness balance the stimuli across electrodes, electrodes were stimulated sequentially at levels indicated to be most comfortable. The listener was instructed to indicate which stimulus intervals needed adjustments in order for all of the intervals to have equal loudness and for all to be at the most comfortable loudness level. Levels were adjusted according to listener responses.

### Melody Scaling

For each trial of the experimental task with either the electric or the acoustic stimuli, listeners heard one stimulus sequence and were told to rate the melody on a visual scale with numbers from 0 to 100 responding to the question “How out of tune is the melody?” For listeners tested in Belgium, the instructions were translated into Flemmish as “Hoe vals klinkt deze melodie?” Listeners could hear the sequence as many times as desired before moving on to the next trial. Four equally spaced descriptors were provided along the rating scale from bottom to top: “in tune” (“helemaal niet vals”), “a little out of tune” (“bijna niet vals”), “out of tune” (“vals”), and “unrecognizable” (“onherkenbaar”).

Three CI users with usable contralateral acoustic hearing (UZA-SSD-M1, UZA-SSD-M11, UZA-SSD-M16) completed the experiment with the pure-tone stimuli (7 Semitone Exponents × 2 Root Notes) before starting the experiment with the electric stimuli (7 Semitone Exponents × 5 Electrodes × 2 Root Notes). Pure tones were tested first to verify that the participants understood the task with the electric stimuli. This may have improved the performance of these listeners with the electric stimuli, but this was not considered problematic, as we wanted to observe the participants’ best performance. NYU-M101 completed the experiment with the electric stimuli before completing half of the pure-tone stimuli (7 Semitone Exponents × 1 Root Note [90 Hz]). Acoustic stimuli were presented only to unimplanted ears. The NH listeners completed the experiment with the pure-tone stimuli before the experiment with the acoustic pulsatile stimuli (7 Semitone Exponents × 2 Center Frequencies × 2 Root Notes). Within each set of stimuli (pure tone, electric, acoustic pulsatile), conditions were fully randomized across trials. Three to five trials were collected per condition for the listeners with CIs depending on time availability, and five trials were collected for each condition for the NH listeners as indicated in Table 3.

**Table 3.** The Number of Trials Collected for Each Listener With Each Type of Stimuli.

Participant	Electric stimuli	Pure-tone stimuli	Acoustic pulsatile stimuli
UZA-SSD-M1	5	4	–
UZA-SSD-M11	4	4	–
UZA-SSD-M16	4	5	–
NYU-M101	5	5	–
NYU-M102	3	–	–
UZA-M13	5	–	–
UZA-M10	5	–	–
UZA-M16	5	–	–
UZA-M17	4	–	–
NH102	–	5	5
NH103	–	5	5
NH104	–	5	5
NH108	–	5	5
NH109	–	5	5
NH114	–	5	5
NH115	–	5	5
NH116	–	5	5
NH117	–	5	5
NH118	–	5	5

## Results

Rank transformations were applied to the ratings from the scaling task prior to statistical testing due to the possibility that the data were not normally distributed (Zimmerman & Zumbo, 1993). One-way repeated-measures analysis of variance was used to examine the effect of semitone exponent on the ranks for each root note or each combination of electrode (or center frequency) and root note.

**Figure 3.**
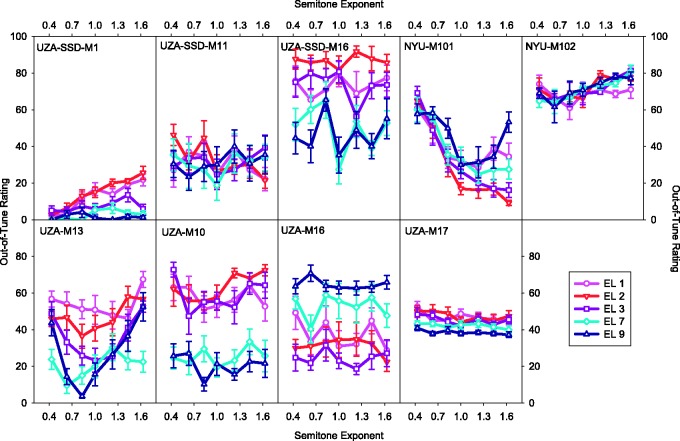
Out-of-tune ratings as a function of semitone exponent for the electric stimuli. Each panel shows ratings of an individual listener with a CI. Each shape and color indicates ratings from a specific electrode. Note that the first four panels of the top row represent participants with usable contralateral acoustic hearing. Error bars indicate ± 1 standard error of the mean.

**Figure 4.**
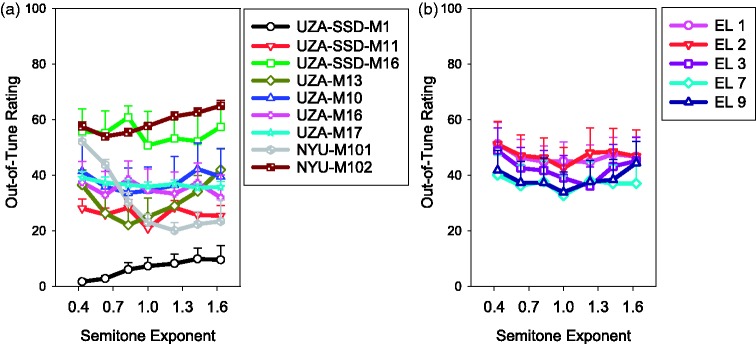
Average out-of-tune ratings as a function of semitone exponent for the electric stimuli. Each shape and color indicates average ratings for each participant (Panel a) or for each electrode (Panel b). Error bars indicate ± 1 standard error of the mean.

Figure 3 shows the results of the scaling task with the electric stimuli as a function of semitone exponent for each electrode tested. Each panel shows the results of an individual CI user. Each data point is an average over the two root notes. Root notes were collapsed in the figures for visual simplicity. No consistent effect of semitone exponent is apparent for any of the electrodes. Figure 4 shows the ratings for the electric stimuli for each listener averaged across electrodes (Figure 4(a)) and for each electrode averaged across listeners (Figure 4(b)), with no apparent effect of the semitone exponent. No significant effect of semitone exponent was found for any combination of electrode and root note, *F*(6, 48) < 1.64, *p* > .15. This was the case also when the first two trials of each condition for each listener were excluded from the analysis on the conjecture that the listeners may have improved on later trials, *F*(6, 48) < 2.11, *p* > .07.

**Figure 5.**
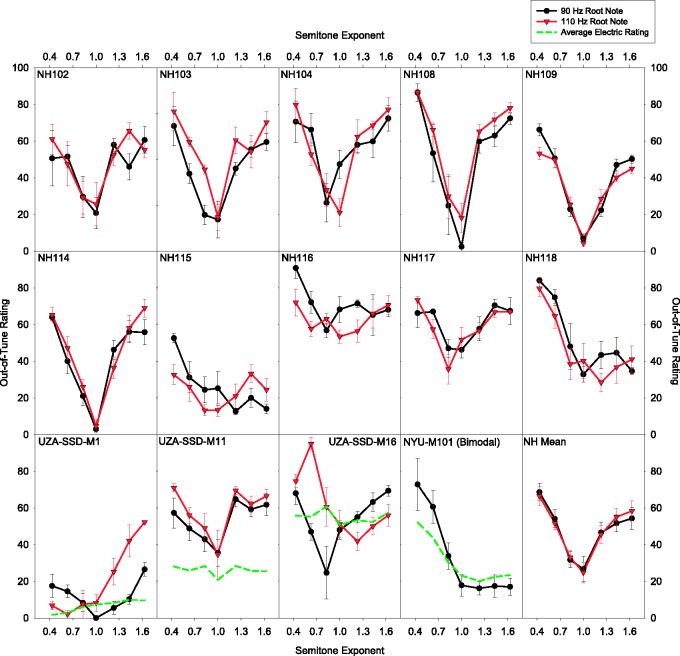
Out-of-tune ratings as a function of semitone exponent for the pure-tone stimuli. Each panel shows ratings from an individual listener. The top two rows consist of participants with NH in both ears. The final panel shows the average across these NH listeners. The participants in the bottom row have a CI in one ear and acoustic hearing in the other. Note that participants UZA-SSD-M1 and NYU-M101 had hearing loss in their acoustic ears. Each shape and color indicates the root notes of the stimuli presented to the acoustic ear. For the participants with a contralateral CI, their average electric out-of-tune ratings are indicated with a dashed green line. Error bars indicate ± 1 standard error of the mean.

In contrast, Figure 5 shows the ratings of the pure-tone stimuli for each of the NH listeners and the four listeners with contralateral CIs. The final panel of Figure 5 shows the average of all of the NH listeners (but not the four listeners with contralateral CIs). For all listeners with the pure-tone stimuli, there was a significant effect of semitone exponent for the 90-Hz root note, *F*(6, 78) = 15.52, *p* < .001, and the 110-Hz root note, *F*(6, 72) = 20.27, *p* < .001. The effect of semitone exponent is highly apparent for the pure-tone stimuli with more extreme out-of-tune ratings occurring with greater compression or expansion of the melody, as expected. For the four listeners with contralateral CIs (UZA-SSD-M1, UZA-SSD-M11, UZA-SSD-M16, and NYU-M101), ratings with the pure-tone stimuli generally follow the expected pattern. A notable asymmetry in ratings is apparent for NYU-M101, who had the most and earliest acoustic hearing loss. For some NH listeners (e.g., NH116, NH117), the melody was always rated as somewhat out of tune (i.e., ratings never neared 0), which may be explained by the listeners’ limited musical experience or tendency to use a limited range of the scale.

**Figure 6.**
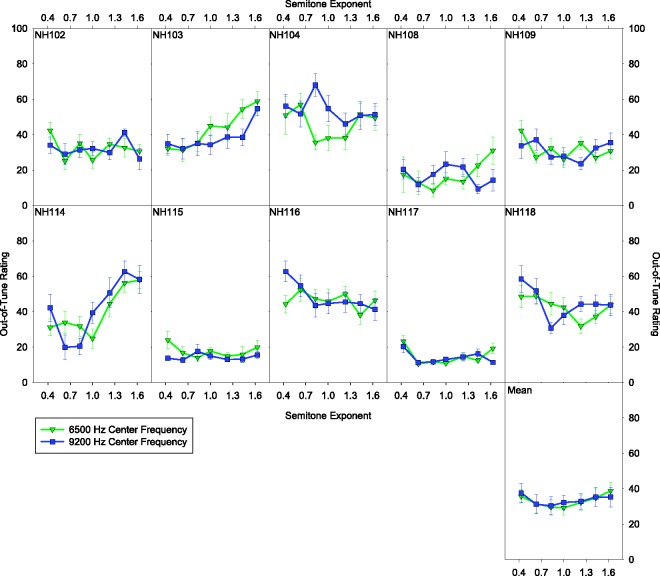
Out-of-tune ratings as a function of semitone exponent for the acoustic pulsatile stimuli. Each panel shows ratings from an individual with NH. The bottom right panel shows the average ratings across listeners. Each shape and color indicates the center frequency of the stimuli. Error bars indicate ± 1 standard error of the mean.

**Figure 7.**
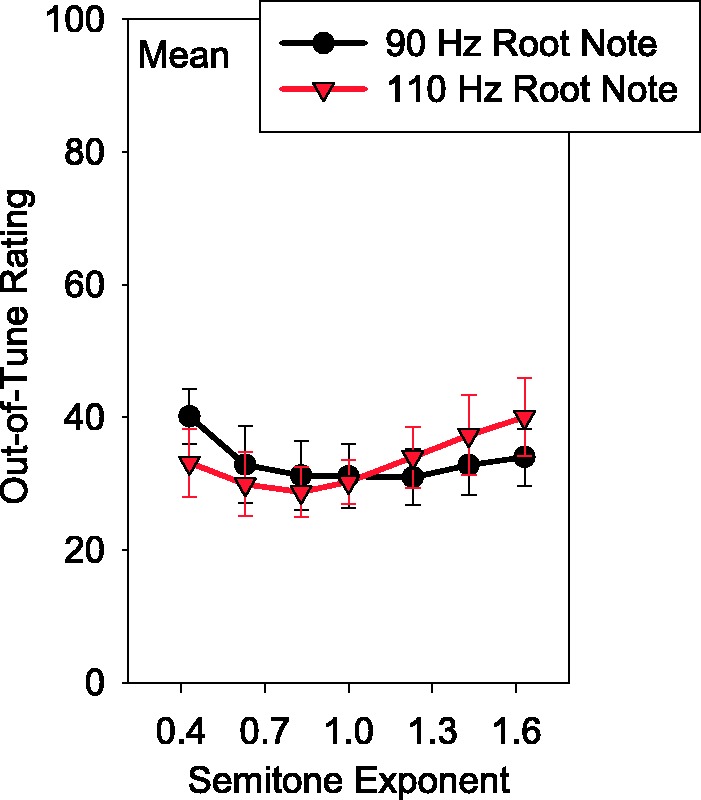
Average out-of-tune ratings as a function of semitone exponent with the acoustic pulsatile stimuli for the NH listeners. The root note of the stimuli is indicated by shape and color. Error bars indicate ± 1 standard error of the mean.

Figure 6 shows the ratings of the acoustic pulsatile stimuli for each of the NH listeners at each center frequency. Each data point is an average across root notes. The last panel of Figure 6 shows ratings for the acoustic pulsatile stimuli averaged across listeners. There was a significant effect of semitone exponent with the 6500-Hz center frequency and 110-Hz root note, *F*(6, 54) = 2.503, *p* = .032. The effect of semitone exponent was not significant for any other combination of center frequency and root note, *F*(6, 54) < 1.57, *p* > .17. Indeed, the results with the acoustic pulsatile stimuli appear more similar to those with the electric stimuli than to the results with the pure-tone stimuli. Figure 7 shows the average ratings of each root note separately. There was a tendency for listeners to rate the 90-Hz root-note stimuli as being more out of tune than the 110-Hz in the compressed condition and vice versa in the expanded condition.

## Discussion

In this study, we examined whether temporal coding provides rational pitch to CI users for melody perception. The use of temporal coding is of interest because preservation of accurate pitch through place coding is difficult with a CI. To investigate rational pitch using temporal coding, we compressed and expanded the melody of a familiar song by raising each semitone to an exponent. The notes were presented using the amplitude-modulation rate of pulse trains from single electrodes. Listener ratings indicating that the melody was more out of tune with greater compression or greater expansion would suggest that the listeners perceived the melody with rational pitch. The same question was examined in NH listeners by manipulating the rate of acoustic pulse trains. The same task was done using pure tones in the acoustic ear of four listeners with CIs as well as with NH listeners to verify that listeners could perform the task. We were also interested in examining whether evidence of rational pitch was more pronounced with apical stimulation than with more basal stimulation, because previous studies suggest advantages of apical stimulation for perception of temporal information (Middlebrooks & Snyder, 2010; Stahl et al., 2016).

With electrical stimulation, listeners on average did not show sensitivity to melody compression or expansion (Figure 4(b)), suggesting that either they received a weak pitch sensation from the temporal coding or there were always some intervals that were perceived as out of tune even in the physically correct condition. This was in contrast to the results with the pure-tone stimuli (Figure 5), with which most of the listeners with contralateral CIs and the NH listeners showed the expected pattern of performance, that is, more out-of-tune ratings with greater compression or expansion of the melody. Because the listeners with CIs in one ear and acoustic hearing in the other ear were tested with the pure-tone stimuli before the electric stimuli, it is unlikely that lack of task comprehension or memory of the song is the reason the CI users did not show sensitivity to melody compression or expansion with the electric stimuli. Rather, it seems the temporal coding did not provide the CI users with sufficient rational pitch (i.e., the pitch was weak). Another explanation is that the temporal coding provided the CI users with rational pitch but the rational pitch was insufficiently accurate. There was also no evidence of greater sensitivity to melody compression or expansion with apical stimulation than with basal stimulation. It is possible that there are advantages to apical stimulation as was found by Stahl et al. (2016) but those advantages do not extend to either the stimuli or the task in the present study. Further research is needed to understand whether and in what contexts apical stimulation is beneficial.

In a few instances, individual listeners with CIs appeared to show sensitivity to melody compression or expansion with some electrodes (Figure 3). For example, the ratings of participant UZA-M13 with electrodes 3 and 9 showed a pattern similar to that of the NH pure-tone ratings. Similarly, the ratings of NYU-M101 with Electrode 9 had a pattern that was similar to that of the NH pure-tone ratings. Otherwise, NYU-M101 tended to rate larger semitone exponents as more in tune, with ratings plateauing for the expanded melodies. This was an atypical pattern yet it suggested NYU-M101 was sensitive to melody compression. Interestingly, this pattern was similar to that of NYU-M101’s ratings with the pure-tone stimuli. This unique pattern may be related to the fact that NYU-M101 had congenital hearing loss (Table 1), which may have limited this participant’s knowledge of the melody of the song. Possibly, the greater sensitivity of both UZA-M13 and NYU-M101 to melody compression or expansion may be related to the fact that they both had taken several years of music lessons (Table 2). In contrast, some of the CI listeners (e.g., UZA-SSD-M16, UZA-M10, UZA-M16) appeared to make judgments about how out of tune the melody was based largely on the stimulating electrode. Potentially, the differences in sound quality between different electrodes influenced the listeners’ ratings (Landsberger et al., 2016). In these cases, the trial-to-trial changes in place of stimulation in the melody scaling task may have provided a much more salient perceptual change than the trial-to-trial changes in melody compression or expansion.

It is worth considering whether limitations in amplitude-modulation rate discrimination may have limited sensitivity to melody compression and expansion in the present study. Rate discrimination was necessary for perceiving the intervals of the song; however, adequate rate discrimination would not necessarily indicate that the listeners perceived the intended intervals since interval discrimination is not as accurate as frequency discrimination (J. H. McDermott, Keebler, Micheyl, & Oxenham, 2010). On average, just-noticeable differences in rate for low-rate amplitude modulations are around 10% for CI users (Kreft et al., 2010; Landsberger, 2008). In the most compressed condition, many notes were likely not discriminable. However, the largest interval was a 34% increase, and there were four other intervals that consisted of changes >10%. In the most expanded condition, intervals were relatively large with 17 out of 20 consisting of changes larger than 10%. In this condition, in which some notes were relatively high in rate, there may have been limitations in rate discrimination for the higher rate notes due to the increase in rate difference limens at higher rates. However, of the 17 intervals exceeding 10%, 12 (root note 110 Hz) to 14 (root note 90 Hz) were below 200 Hz and likely provided discriminable temporal pitch. In the physically correct condition, 17 out of the 20 intervals consisted of changes in amplitude-modulation rate that were >10%. Furthermore, in the physically correct condition, rates did not exceed 180 Hz (90 Hz root note) and 210 Hz (110 Hz root note), suggesting that listeners likely had access to discriminable temporal pitch for these intervals. Therefore, many of the notes within trials were likely discriminable. However, this does not necessarily indicate that the intervals were discriminable across different melody compression or expansion conditions, which would be necessary for the listeners to make judgments about tuning.

The performance of the CI users in the present study was poorer than what might be expected based on the performance of the three CI users examined by Pijl and Schwarz (1995a, 1995b). One reason for this may be that Pijl and Schwarz (1995a, 1995b) only examined a small sample of listeners and those listeners were chosen based on good performance on a melody recognition task. Thus, their results are unlikely to be generalizable. A second reason may have to do with the findings of Pijl and Schwarz (1995a, 1995b) as well as H. J. McDermott and McKay (1997) that suggest that perception of musical intervals in CI users depends on the rate used to encode the interval and the size of the interval. Two of the three listeners examined by Pijl and Schwarz (1995b) selected larger intervals than expected for a musical fifth at a base rate of 81 pps and all three selected smaller intervals as the base rate increased to 163 and then to 326 pps. H. J. McDermott and McKay (1997) found that one musically trained CI user could identify small musical intervals when pitch was encoded with pulse rate or amplitude-modulation rate (for rates 100–200 Hz) but underestimated larger musical intervals. These findings suggest that with melodies, which are composed of multiple rates and multiple intervals, there may always be some intervals that sound out of tune even if the melody is physically correct. Thus, the listeners in the present study may have performed worse than those in Pijl and Schwarz (1995a, 1995b) because of the greater variety of intervals that composed the stimuli. The idea that all versions of the song may have sounded out of tune matches the report of some of the CI users that some intervals could sound in tune while others did not. Similar reports have been made by other CI users in other melody perception studies (Eddington et al., 1978; Luo et al., 2014). However, this does not explain the performance of participant UZA-SSD-M1 who reported the majority of conditions as sounding in tune. This participant may have had difficulty identifying the compressed and expanded versions of the song as being out of tune if memory of the song dominated perception. The fact that the rhythm of the song was intact could have facilitated this possibility. We would expect this to be the case if the temporal pitch was particularly weak for this participant.

When the stimuli consisted of acoustic pulse trains, the NH listeners showed sensitivity to melody compression or expansion at the lowest center frequency and highest root note (Figure 6). However, the effect of melody compression or expansion was far weaker than what was observed with the pure-tone stimuli (Figure 5). It is possible that a significant effect of melody compression or expansion was only found at the lowest center frequency and highest root note due to resolvability of the harmonics in this condition by the peripheral auditory system. While previous research suggests that this was unlikely for the rates in this condition (Carlyon & Deeks, 2002), it is possible the highest rate was resolvable. Furthermore, there was a tendency for listeners to rate the compressed stimuli as more out of tune with the lower root note and to rate the expanded stimuli as more out of tune with the higher root note (Figure 7). This is consistent with the idea that listeners were relying on the highest notes of each trial to do the scaling task, judging low highest notes (the compressed, low root-note condition) and high highest notes (the expanded, high root-note condition) as out of tune as opposed to relying on rational pitch. It is also possible that the NH listeners had a sense of how out of tune the song was based on some aspect of the sound other than rational pitch such as the buzzing of the amplitude modulation. It has been found that listeners can use changes in loudness and brightness to assist with melody identification (J. H. McDermott, Lehr, & Oxenham, 2008). Thus, possibly the listeners could use the extent of changes in the buzzing from note to note as a cue for how out of tune the song was. However, if listeners were using this type of cue, it was a weak cue resulting in performance unlike that with pure tones. The weak effect of melody compression and expansion suggests, like with the electric pulsatile stimuli, NH listeners have difficulty using temporal coding to judge musical intervals. In some instances, for both the NH listeners and the CI users with contralateral acoustic hearing (e.g., NH117, UZA-SSD-M11), the pulsatile stimuli were rated as more in tune than the pure-tone stimuli. It is possible that the participants were conceptually using two different scales for the pure-tone and pulsatile stimuli. This could be due to the fact that the pure-tone and pulsatile stimuli were tested at separate times and sounded different. Furthermore, if rational pitch was weak with the pulsatile stimuli, it is possible that listeners could never detect the melody as being out of tune. Thus, some listeners may have had a tendency to provide highly in tune ratings with the pulsatile stimuli.

Other studies have more clearly demonstrated that NH listeners can use the pitch obtained from temporal coding for musical interval perception; however, NH listeners do so with imperfect performance (Burns & Viemeister, 1981; Houtsma & Smurzynski, 1990; Moore & Rosen, 1979). NH listeners can recognize melodies above chance with temporal coding alone, but performance is not as good as when stimuli are pure tones or complex tones with resolvable harmonics (Burns & Viemeister, 1981; Moore & Rosen, 1979). Furthermore, musically trained NH listeners identify and match musical intervals above chance, and errors tend to be within two semitones (Burns & Viemeister, 1981; Houtsma & Smurzynski, 1990). Possibly, the NH listeners in the present study would have done better with wider band stimuli, which have been found to result in more accurate interval identification (Houtsma & Smurzynski, 1990).

In summary, CI users demonstrated an inability to judge the degree to which a familiar song was out of tune when the melody was presented through amplitude-modulation rate. This was shown despite the finding that three of the four CI users who had contralateral acoustic hearing generally rated the song as more out of tune with greater physical mistuning when the stimuli were presented to their acoustic ear with pure-tone stimuli. Similarly, NH listeners were less sensitive to mistunings when the melody was encoded in the rate of acoustic pulse trains compared with the frequency of pure tones. The results suggest that CI and NH listeners have difficulty using temporal pitch for rational pitch (i.e., to perceive musical intervals) in the absence of place pitch cues. Difficulty perceiving rational pitch with temporal coding was found at basal and apical stimulation sites for the CI users, providing no evidence of a difference between apical and basal stimulation sites for temporal pitch processing. Further research is needed to investigate whether there are conditions in which temporal coding can be used to provide rational pitch to CI users. Potentially, rational pitch with temporal coding could be improved with the use of synchronized modulations across multiple electrodes (Milczynski, Wouters, & van Wieringen, 2009). The effect of concurrent manipulations in place and rate of stimulation and listener training are also areas to explore in their effect on rational pitch with temporal coding.
